# Reply to the letter to editor

**DOI:** 10.1080/2090598X.2020.1833480

**Published:** 2020-10-28

**Authors:** Rabea G. Omar, Mostafa M. Khalil, Hesham Alezaby, Ahmed Sebaey, Hammouda Sherif, Ahmed Mohey

**Affiliations:** Department of Urology, Faculty of Medicine, Benha University, Benha, Egypt

First of all the title of our paper is evaluation of erectile function after anastomotic vs subsitution urethroplasty for bulbar urethral stricture. We evaluated erectile function only and not sexual function, as the erectile function is the most important aspect of all patient sexual satisfaction.

There was no history of diabetes or any drugs affecting erectile function in the study groups and all patients had normal erection before urethroplasty, and any patient with ED was excluded.

We used the IIEF 15 score ‘EF Domain’ only for evaluation of patient erectile function pre- and postoperatively and did not use others to fix one method for evaluation of all patients, as we evaluated the urethroplasty technique not the score. Patients with ED need penile duplex ultrasonography.

Also, we acknowledge the small sample size of the study groups as these cases are not common enough to do on large sample size of patients.

